# Editorial: Psychosocial factors, mental comorbidities and related biomarkers interacting with the onset and course of somatic diseases

**DOI:** 10.3389/fpsyt.2026.1927855

**Published:** 2026-09-15

**Authors:** Gloria-Beatrice Wintermann, Michael Noll-Hussong

**Affiliations:** 1 Department of Psychotherapy and Psychosomatic Medicine, Faculty of Medicine and University Hospital Carl Gustav Carus, TUD Dresden University of Technology, Dresden, Germany; 2 Psychosomatic Medicine and Psychotherapy, Day-Hospital Westend, Munich, Germany

**Keywords:** consultation-liaison psychiatry, disease coping, life-style behavior, mental comorbidity, psycho-somatic interaction, somatic disease

## Bidirectional mind–body interactions and moderating factors

Facing the ageing of the population, medical progress, social and epidemiological development, the number of patients suffering from chronic somatic diseases and mental disorders will grow in the future (1, 2). Former projections of mortality and burden of disease by cause until 2030 predicted a dramatic shift from communicable to non-communicable diseases, such as ischaemic heart disease and unipolar depressive disorders, which are among the two leading causes of years lived with disability (3). Above, mental disorders represent a substantial global health burden (4). According to the Global Burden of Disease Study 2019, mental disorders accounted for 14.6% of all years lived with disability worldwide, emphasizing the need for integrated approaches, addressing both somatic and psychological aspects of chronic diseases (2). Comorbid mental disorders may complicate help-seeking, increase the chance for the manifestation, exacerbation and complications of somatic diseases, and lead to an overall diminished quality of life (5–8). They may negatively affect the vulnerability for infectious diseases and their long-term consequences (9, 10). Finally, the patients´ treatment adherence may be reduced, accelerating disease progression, and increasing the risk for mortality (11, 12).

Several publications in the current topic have focused on the complex relationship between chronic somatic diseases and mental comorbidities. They provide evidence about relevant moderator variables in this complex, bidirectional psycho-somatic interaction, drawing practical implications to optimize treatment, identify at-risk population, and offer adjuvant psychosocial interventions as well as integrated care models in these patients. The findings of these studies highlight Influencing factors such as lifestyle behavior, disease coping, emotional awareness, resilience, and social support – which may have a major impact on the course of somatic illnesses and comorbid mental disorders.

## Lifestyle behavior and disease coping as moderators of clinical outcomes

In the cross-sectional study by Ledermann et al., the results showed that erectile dysfunction (ED) was common among implantable cardioverter defibrillators (ICD) patients. In patients with more externally oriented thinking (high preoccupation) about their illness, certain aspects of alexithymia were linked to more severe erectile problems. The study emphasizes the importance of considering psychological support in order to improve emotional expression, and addressing sexual health as part of comprehensive care for ICD patients.

In the cross-sectional study by Wang et al., data from the U.S. National Health and Nutrition Examination Survey (NHANES) database were analyzed according to the impact of depression rating on the risk for chronic diarrhea, depending on the alcohol drinking status. They found that the prevalence for depression with chronic diarrhea was more than twice higher in the drinking than in the non-drinking population. The findings underscore the role of psychological factors and lifestyle behaviors in gastrointestinal symptoms and the gut–brain–behavior interaction. Clinicians should be aware of the impact of alcohol consumption on both the mental and digestive health. Drinking habits of patients with depression and chronic diarrhea should be considered in the treatment plans.

## Causal perspective on the psycho-somatic interaction

In a Mendelian randomization study conducted by Yang et al. in individuals of European ancestry, genome-wide association data comprising 108 single nucleotide polymorphisms were used to investigate the causal relationship between severe mental disorders, including major depressive disorder and schizophrenia, and the risk of sepsis and sepsis-related mortality. The authors found that genetically predicted major depression, but not schizophrenia, was associated with an increased risk of sepsis, while no significant association was observed for sepsis mortality. These findings provide evidence for a potential causal link between major depression and susceptibility to severe infections, possibly mediated by pro-inflammatory cytokine dysregulation and alterations in T-cell subsets. This may contribute to an increased vulnerability to infection and sepsis in patients with major depressive disorder and underscores the importance of heightened clinical awareness in this population.

## Psychological resources and well-being

Zhang et al. examined perceived uncertainty and anxiety arising from unpredictable aspects of chronic, potentially life-threatening conditions in a sample of patients with Moyamoya disease, a rare cerebrovascular disorder characterized by recurrent ischemic and hemorrhagic strokes. In this cross-sectional study, the authors investigated the influence of sociodemographic and psychosocial factors, such as resilience (defined as the ability to adapt to and cope with adverse life events) and perceived social support, on illness uncertainty. The findings indicated that male gender, higher educational attainment, greater resilience, and higher perceived social support were associated with lower levels of illness uncertainty. These results underscore the importance of tailoring psychosocial interventions for this patient population, in order to improve psychological well-being, overall quality of life, and disease coping.

In their mini-review, Zuccarella-Hackl et al. summarize the current evidence on the relationship between positive psychological well-being and cardiovascular health. They discuss both hedonic and eudaimonic dimensions of well-being, including positive affect, optimism, self-acceptance, and purpose in life. According to the authors, higher levels of positive psychological well-being are associated with a reduced risk of cardiovascular disease and more favorable cardiovascular outcomes. These associations are thought to be mediated by healthier lifestyle behaviors, more effective coping strategies, and reduced activation of stress-related neuroendocrine, autonomic, and inflammatory pathways.

In their systematic review, Liu et al. examined the extent of benefit finding among adults living with somatic non-communicable chronic diseases (SNCDs). Benefit finding refers to the perception of positive psychological changes resulting from the experience of living with a chronic illness. The findings demonstrate that benefit finding is associated with adaptive psychological response to chronic illness, more effective coping, improved psychological well-being, and better health-related quality of life. The authors conclude that promoting benefit finding may complement traditional medical care.

## Health services and consultation-liaison psychiatry during the COVID-19 pandemic and post-COVID-19 symptoms as contemporary challenge

In a retrospective study, Prina et al. examined the impact of COVID-19 pandemic-related restrictions on psychiatric and psychological consultation-liaison (CL) services in a general hospital in Verona. The authors observed an increase in psychiatric and psychological consultations during the early phase of the pandemic, particularly among outpatients. However, this was followed by a reduction in consultation rates during periods of lockdown, with a smaller drop during the intermediate restriction phase. These findings demonstrate the persistent psychological burden of patients and the need for adapted mental health care delivery models under pandemic conditions, in order to ensure continuous access to psychiatric and psychological support.

In their longitudinal study, Muller et al. investigated the course of neuropsychological health in patients recovering from workplace-acquired COVID-19 at 6 and 12 months after inpatient rehabilitation. A substantial proportion (about one-third) continued to experience persistent symptoms, including fatigue, cognitive impairment, anxiety, and depressive symptoms 12 months after rehabilitation. The authors conclude that continuous assessment of cognitive and psychological functioning is essential to identify patients with persistent impairments and to provide targeted, multidisciplinary therapeutic interventions that support recovery and improve quality of life in post-COVID patients.

## Summary and outlook

As the burden of non-communicable disease continues to grow, addressing mental comorbidities as an integral component of chronic disease management is not optional—it is essential. The studies included in the present Research Topic give profound insight in the complex interaction between somatic and mental health conditions. In the future, more adequately powered, externally valid, multi-method studies (complementing self-report with expert-based and biological data) are needed to investigate the impact of sociodemographic, clinical and psychosocial variables on the manifestation and course of somatic illnesses and mental comorbidities. Longitudinal studies with long-term follow-ups, controlling for relevant confounders, using standardized and valid assessment methods, and focused interventional studies would help to better draw conclusions about causal relationships. Besides risk factors (such as age, gender, educational level, severity of disease) also protective factors should be more investigated (such as benefit finding, resilience). Above, research should unravel clinical and organizational factors that influence the accessibility of adjuvant psychosocial services.

We hope this Research Topic stimulates further research and informs clinical practice, ultimately improving outcomes for patients who are challenged by the complex relationship between chronic somatic diseases and mental comorbidities.
